# Impact of the Low-Price Medicine Policy on Medicine Supply in China: An Interrupted Time-Series Analysis

**DOI:** 10.3389/fphar.2021.621307

**Published:** 2021-02-05

**Authors:** Mingyue Zhao, Ali Hassan Gillani, Duan Ji, Zhitong Feng, Yu Fang, Caijun Yang

**Affiliations:** ^1^ Department of Pharmacy Administration and Clinical Pharmacy, School of Pharmacy, Xi’an Jiaotong University, Xi’an, China; ^2^ Center for Drug Safety and Policy Research, Xi’an Jiaotong University, Xi’an, China; ^3^ School of Accounting, Shandong University of Finance and Economics, Ji’nan, China

**Keywords:** medicine supply, interrupted time series analysis, low‐price medicine policy, medicine price, medicine shortage

## Abstract

**Objectives:** The primary objective of the study was to assess the impact of the Low-Price Medicine Policy (LPMP) on the supply of low-price medicines (LPMs) in China. The secondary objective of the study was to describe the supply situation of LPMs from 2005 to 2018.

**Methods:** The LPMP was launched in the third quarter of 2014 (2014Q3). An interrupted time series analysis was used to evaluate the impact of LPMP on the supply of LPMs in China. Ordinary least squares and Poisson regression models were utilized to estimate the effect of LPMP on LPMs’ supply growth rate and the number of supplied LPMs. All the LPMs were divided into two subgroups: intermittent supply and continuous supply. The trend and level changes of the quarterly average growth rate and number of quarterly supplies for different LPM groups were analyzed from 2005 to 2018.

**Findings:** For the quarterly average growth rate, before the intervention, a significant increasing trend was observed in the total group and the continuous supply subgroup; after the introduction of LPMP, the increasing trend was ceased and a significant decrease in the trend and level was noted for both the total group (trend coefficient: *β*_*3*_ *=* −0.0132, *p* < 0.01; level coefficient: *β*_*2*_ = −0.1510, *p* < 0.05) and the continuous supply subgroup (trend coefficient: *β*_*3*_ = −0.0133, *p* < 0.01; level coefficient: *β*_*2*_ = −0.1520, *p* < 0.05); whereas it had no significant effect for intermittent supply subgroup. For the number of quarterly supplies, after the intervention of LPMP, decline of the supply number was observed (trend coefficient: *β*_*3*_ = −0.0027, *p* < 0.001; level coefficient: *β*_*2*_ = −0.0584, *p* < 0.001); whereas the LPMP was associated with an upward trend and level (trend coefficient: *β*_*3*_ = 0.0715, *p* < 0.001; level coefficient: *β*_*2*_ = 0.174) for the intermittent supply subgroup.

**Conclusion:** For most of the LPMs, LPMP did not meet the goal of stimulating LPM production. However, for severely shortage medicines (the intermittent supply subgroup), the effect of LPMP was positive. Comprehensive policies rather than just deregulating medicine price should be introduced to alleviate the situation of medicine shortage in China.

## Introduction

Medicine shortage is a complex challenge to health systems worldwide. The International Pharmaceutical Federation (International Pharmaceutical Federation, 2020) describes it as “a medicine supply issue requiring a change that impacts patient care and needs the use of an alternative agent.” Medicine shortage has affected all health system stakeholders, especially patients. It directly increases patients’ risk and disease burden by restricting access to medicine, giving rise to increased medication errors and other unsafe practices (Gray and Manasse, 2012; Casassus, 2015). Although shortages may disrupt any class of medicines, studies have shown that low-price generic medicines face a higher risk of shortage than other medicines because of their lower prices (Dave et al., 2018).

China experiences severe medicine shortages, and most of the affected medicines are low-price generics (Fan et al., 2018; Wu et al., 2016; Yang et al., 2018). One study identified 139 medicines that faced shortages in Shaanxi Province, northwestern China, in 2016, and 62.6% of these were low-price medicines (LPMs; Cai et al., 2017). Liu (2007) empirically examined the relationship between medicine shortage and mandatory price reduction in China from 1997 to 2007. The study showed that the number of medicines in short supply increased immediately after each price reduction. Manufacturers are less motivated to produce low-price drugs because they are less profitable than expensive drugs. As a result, manufacturers have tended to shift their attention to producing more profitable medicines, which are mainly the expensive ones (Zhang et al., 2012). Thus, low prices for medicines are considered one of principal causes of medicine shortages in China (Yang et al., 2016; Dave et al., 2018). From the perspective of the users of medical services, patients are prescribed high-price medicines that are popular among doctors in China because they bring higher revenue (Yu et al., 2010). Thus, the mark-up policy^1^ could easily lead to a benefit chain among doctors, pharmaceutical enterprises, and sales representatives in China (Yang et al., 2016). Furthermore, the population of China increased by approximately 8% during the period of this study (National Bureau Statistics, 2019). The population growth would certainly increase the demand for medicine. This implies that there will be medicines shortages if their supply does not increase over time. Therefore, researchers and policy makers should pay more attention to increased production of low-price medicines, especially those that are in short supply according to media reports.

To address the shortage problem of low-price medicines, the government promulgated the Low‐Price Medicine Policy (LPMP) in 2014, which raised the price cap^2^ of LPMs. A total of 533 LPMs, including 283 low-price chemical medicines (LPCMs) and 250 low-price traditional Chinese medicines (LPTCMs), were selected for deregulation. Manufacturers can now freely set the price for these 533 LPMs, as long as the daily cost of chemical medicines and traditional Chinese medicines is no more than three and five Chinese Yuan (CNY), respectively. All of the LPMs that can meet basic clinical needs and reduce the burden on patients are included in the Urban Employee Basic Medical Insurance (UEBMI) list^3^. In addition to the 2014 policy, a new round of pharmaceutical price reform was introduced in China in 2015, referred to as the “2015 reform” (National Development and Reform Commission, 2015). This reform deregulated the price of medicines covered by the UEBMI list, except for some anesthetics and psychotropic medicines. Note that all of the LPMs are covered by the UEBMI list. The reform has not only relaxed the price regulation of LPMs but has also freed the other medicines covered by the UEBMI list from price control. The aim of this reform is to construct a market-driven pricing mechanism for pharmaceuticals. Thus, medical resources could be efficiently allocated and the supply and-demand imbalance could be solved. Therefore, the 2014 LPMP can be regarded as a pilot initiative for the 2015 LPM reform.

The effect of the LPMP on the prices and procurement volumes of LPMs has been the subject of several studies. For example, using the annual data of LPMs in different provinces of China, Zhang, (2016), Song, (2018), and Wang and Wu (2019) found the average LPM price increase in 2015 and 2016. Guan et al. (2018) proved, by using monthly data from January 2012 to July 2015, that the prices of LPMs increased after the LPMP. Rong et al. (2020) evaluated the effect of the LPMP on the prices and procurement volumes of LPMs, using monthly procurement data from hospitals in Shandong province from March 2014 to February 2017. However, there seems to be no study to date evaluating the role of the policy in increasing the supply of LPMs at the national level, which is the main goal of the policy. Therefore, the primary objective of this study is to assess the impact of the LPMP on the supply of LPMs in China. Its secondary objective is to study the supply situation of LPMs in China from 2005 to 2018.

This paper empirically investigates the impact of LPMP on the supply of LPCMs from 2005 to 2018. Both the 2014 LPMPs and the 2015 reform could affect the supply of LPMs. We chose 2014Q3 as the intervention time point because the 2014 LPMP can be regarded as a pilot initiative for the 2015 reform and this study covers only LPMs, whose prices were deregulated for the first time in 2014. The growth rate of medicine supply and the number of supplied medicines are evaluated to study the policy effect on medicine supply. We derive two testable hypotheses for our empirical study. Our first hypothesis is that the supply growth rate of LPCMs is more likely to increase after the LPMP launch in China. The second hypothesis is that the number of LPCMs is more likely to remain the same or increase after the policy intervention. However, our studies showed that both the growth rate and number of medicines supplied decreased after the policy was implemented. Therefore, we concluded that the policy failed to increase the supply of LPMs in the market. In this paper, we present a detailed discussion about the causes of the policy failure.

We found some interesting results by dividing the LPMs into two subgroups: a continuous-supply subgroup and an intermittent-supply subgroup. LPMs in the intermittent-supply face severe shortage, and most of the medicines have captured media attention. However, LPMs in the intermittent-supply subgroup, which are mainly emergency rescue medicines, changed from a decreasing trend in the number of medicines supplied before the policy to an increasing trend after the policy, supposedly because of the fixed-point production mechanism established by the government.

## Materials and Methods

### Data and Sample

Data were collected from the Comprehensive Economic, Industry and Corporate database (CEIC) which is a national macroeconomic, regional economic, industry economic, and thematic time-series database. The data on the pharmaceutical sector in the CEIC were extracted from the Statistics Survey of the Pharmaceutical Industry of China Medical Statistics Network, which is approved by the National Bureau of Statistics. The medicines from CEIC used in our study are certain specific formulations (https://insights.ceicdata.com/Untitled-insight/myseries). The medicine supply information provided by the CEIC includes generic name, quarter, and supply volume per quarter (statistical unit: ton).

We selected 283 LPCMs from the LPM list as the target medicines in this study because LPTCM supply information was not available in the CEIC. Of the 283 LPCMs, 143 medicines were not recorded by the CEIC. According to the rules of the reporting system, this implied that these medicines were no longer in production. Of the remaining 140 LPCMs, 15 were excluded from this study because of incomplete records. Therefore, the final sample comprised 125 LPCMs from 2005 to 2018, or 56 periods (14 years*4 quarters) in total. We used medicine-quarter as our observation unit (statistical unit: ton/quarter).

### Outcome Indicators

The shortage caused by lack of production enthusiasm was regarded as the main problem faced by LPMs in China. Policies to promote the production and supply of LPMs were implemented in China. To evaluate the effect of the policy, supply related indicators were selected as the main indicators in our study. We chose two parameters to reflect the trends and the changing process of medicine supply: supply growth rate and number of medicines supplied.

We define *g*_*it*_ as the growth rate in the supply of medicine *i* in period *t*, which can be calculated according to the following equation:

$${\text{g}}_{\text{it}}=\left({\text{S}}_{\text{it}}-{\text{S}}_{\text{i}1}\right)/{\text{S}}_{\text{i}1}$$

where *S*_*i1*_ is the total volume of medicine *i* supplied in the first period and *S*_*it*_ is the total volume of medicine *i* supplied in the *tth* period. Here, a statistical period is one season (quarter). The first indicator is *g*_*t*_, the quarterly average supply growth rate at time *t* for the 125 LPCMs:

$$g_{\text{t}}=\frac{1}{\text{n}}\sum_{i=1}^{n}{\text{g}}_{\text{it}}$$

where *n* is 125, the total number of LPCMs.

It is better to use a parameter such as growth rate to describe the relative increase or decrease in the supply of each medicine. A few points are noteworthy. First, the production volume is different for each medicine. Second, in our article, the quarterly growth rate is defined as the rate of growth over the first quarter. The growth rate correctly represents the rate of increase in the volume of supply. This method can identify not only the immediate effect but also the trend effect.

The second variable is *N*_*t*_, the number of LPCMs supplied at time *t* (of the 125 LPCMs). Here, the growth rate *g*_*t*_ reflects how much the manufacturer produces, and *N*_*t*_ indicates the production strategies of pharmaceutical companies (whether the companies invest to produce the medicine) from a long-term perspective.

### Statistical Analysis

During the entire time period, some medicines were supplied continuously, whereas some were not. Thus, we divided the sample into two subgroups: intermittent supply and continuous supply. The intermittent-supply subgroup consists of medicines for which the volume of supply was zero for more than half of the study period, before the LPMP was launched. There were 38 periods before the launch of the LPMP in 2014Q3. A medicine is included in the intermittent-supply subgroup if it was supplied for less than 19 quarters before LPMP; otherwise, it is included in the continuous-supply subgroup. Most of the medicines from the intermittent group are in short supply according to media reports (CCTV, 2011; China Development Gate, 2018). That is, the severe medicine shortage in the intermittent group has captured public attention. Therefore, we concluded that these medicines face severe shortage, which is not yet formally defined in China. Furthermore, the seasonal effect is found in the data of supplied number. Using the average value of the current point and previous three data points to present current point value, seasonal effect was modified.

Many researchers consider interrupted time-series (ITS) analysis as the strongest quasi-experimental design to evaluate the longitudinal effects of interventions (Cook and Campbell, 1979; Rashidian et al., 2013). In this study, the ITS model is utilized to estimate changes in growth rate trends as well as the number of medicines after the implementation of the LPMP. The date of LPMP launch in China (2014Q3) was regarded as the intervention time point. Therefore, two segments with one interruption point were constructed. The analysis used the following model:

$${\text{g}}_{\text{t}}\left({\text{N}}_{\text{t}}\right)={\text{β}}_{0}+{\text{β}}_{1}{\text{*time}}_{\text{t}}+{\text{β}}_{2}{\text{*Intervention}}_{\text{t}}+{\text{β}}_{3}{\text{*Tai}}_{\text{t}}+{\text{ε}}_{\text{t}},\;$$

where *g*_*t*_ and *N*_*t*_ are the independent outcome variables (supply growth rate and supply number) at time *t*, respectively. *Time*_*t*_ is a continuous variable defined as the number of periods at time *t*. *Intervention*_*t*_ is an indicator of whether time *t* occurs before (*Intervention*_*t*_ = 0) or after the intervention (*Intervention*_*t*_ = 1). *Tai*_*t*_ represents the number of periods after the intervention at time *t*; for the time before the intervention, *Tai*_*t*_ = 0. *β*_0_ is a constant. *β*_1_ represents the slope during the pre-intervention period. *β*_2_ and *β*_3_ are the changes in the intercept and slope, respectively, from the pre-to the post-intervention period. *ε*_*t*_ is an error term representing the variability not explained by the model.

Ordinary least squares regression was utilized to estimate the effect of the policy intervention on the supply growth rate. Poisson regression was utilized to estimate the effect of the policy intervention on the number of medicines supplied per quarter. The Durbin-Watson statistic was used to test for serial autocorrelation of error terms in the regression models. This involved testing for serial correlation by assuming a first-order autoregressive correlation structure. The Breusch-Pagan statistic was utilized to check for heteroscedasticity in the residuals, and robust regression was adopted to correct it, if found. All analyses were performed using Stata 15.0 (Stata Corporation, College Station, TX, United States).

## Results

### Supply Situation

Table 1 reports medicine supply information for different diseases according to the World Health Organization’s Anatomical Therapeutic Chemical Classification. Our sample covered 11 disease classes. More than 20 medicines were supplied for the top-four diseases (antiinfectives for systemic use, alimentary tract and metabolism, cardiovascular system, and nervous system), much more than those for the other seven diseases. Only four disease classes required intermittently supplied medicines, and the maximum number of medicines supplied was five.

**TABLE 1:** The number of LPCMs for different kinds of dieases and there distribution in each group.

Medicine classification	Total group	Continuous supply subgroup	Intermittent supply subgroup
Antiinfectives for systemic use	31	30	1
Alimentary tract and metabolism	22	20	2
Cardiovascular system	20	15	5
Nervous system	20	20	0
Blood and blood forming organs	6	6	0
Respiratory system	6	5	1
Dermatologicals	5	5	0
Genito urinary system and sex hormones	4	4	0
Antiparasitic products, insecticides and repellents	4	4	0
Sensory organs	4	4	0
Musculo-skeletal system	3	3	0
Total	125	116	9

**FIGURE 1:**
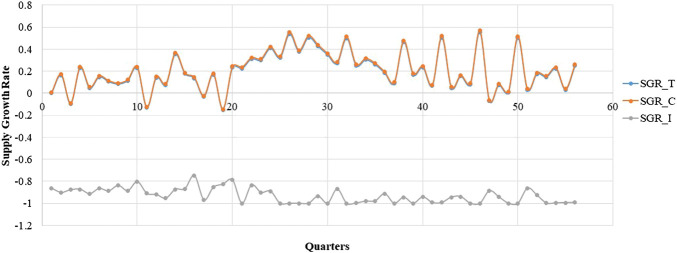
The trend in the quarterly average supply growth rate of LPCMs in China from 2005 to 2018.

**FIGURE 2:**
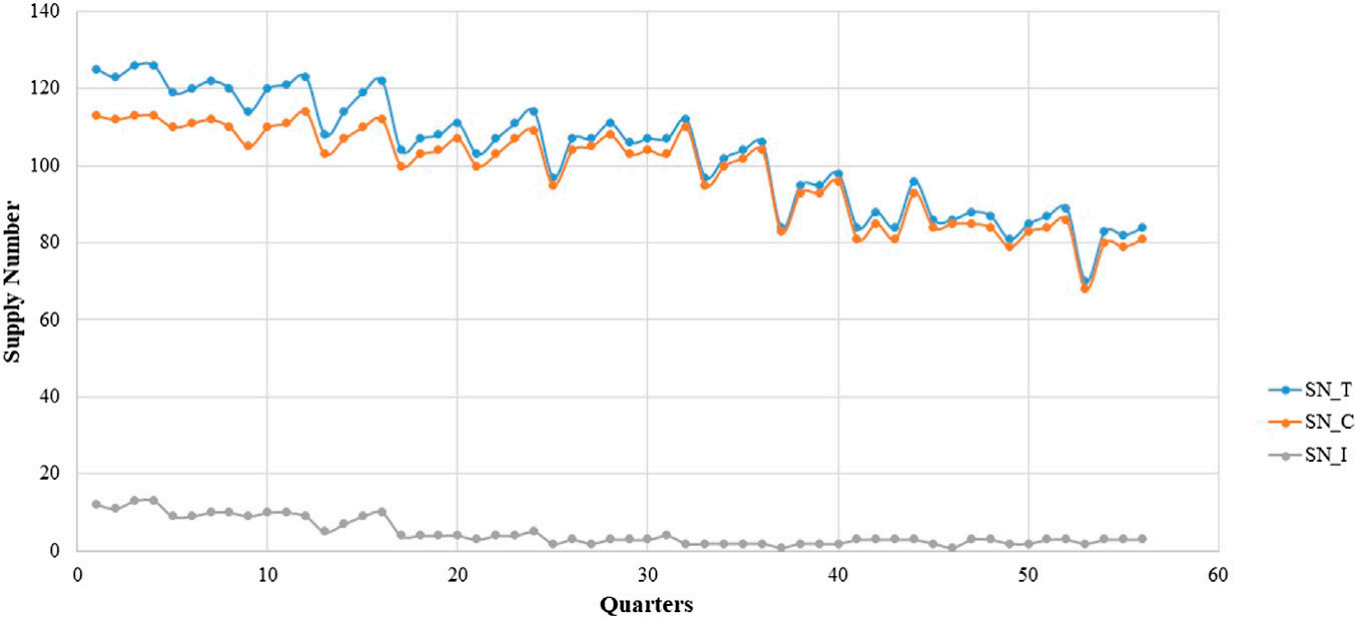
The trend in the quarterly supply number of LPCMs in China from 2005 to 2018.

The box diagram analysis of the quarterly average growth rate shows outliers in our sample, which we needed to correct. The outlier in a certain quarter was replaced with the mean of the corresponding values in the same quarter of the previous year and the next year. The outliers needed to be corrected before seasonal modification. Figures 1, 2 show the quarterly average growth rate and number of medicines supplied quarterly in the continuous- and intermittent-supply subgroups, respectively, from 2005 to 2018. Figures 1, 2 show that the quarterly average growth rates and number of supplied medicines per quarter in the continuous-supply subgroup always exceed those in the intermittent-supply subgroup from 2005 to 2018. As the intervention takes place halfway, we separate the data into pre-intervention and post-intervention periods. From Table 2, we see that both mean growth rates and numbers of medicines supplied decrease after the intervention.

**TABLE 2:** Descriptive statistics of two indicators (*N* = 56 quarters).

Indicators	Total group		Continuous supply subgroup		Intermittent supply subgroup	
	Pre-intervention	Post-intervention	Pre-intervention	Post-intervention	Pre-intervention	Post-intervention
Supply growth rate	0.21 (0.17)	0.18 (0.18)	0.22 (0.17)	0.19 (0.18)	−0.91 (0.07)	−0.97 (0.04)
Supply number	111.29 (9.66)	86.28 (6.24)	105.47 (6.54)	83.72 (6.24)	5.82 (3.72)	2.56 (0.62)

It is obvious that large fluctuations occurred in the continuous-supply subgroup from 2005 to 2018 (Figure 1). Most of the quarterly average growth rates for the continuous-supply subgroup are positive. However, the quarterly average growth rate in the intermittent-supply subgroup is negative, remaining close to -1 in the immediately preceding years. The mean number of supplied medicines in the total group, continuous-supply subgroup, and intermittent-supply subgroup are 103.25 ± 1.95, 99.73 ± 1.75, and 2.52 ± 0.33, respectively. The decreasing trend in the number of medicines is obvious in the total group and continuous-supply subgroup. For the intermittent-supply subgroup, however, the trend in supplied numbers shows a smooth change (Figure 2).

### Influence on the Supply Growth Rate

**FIGURE 3:**
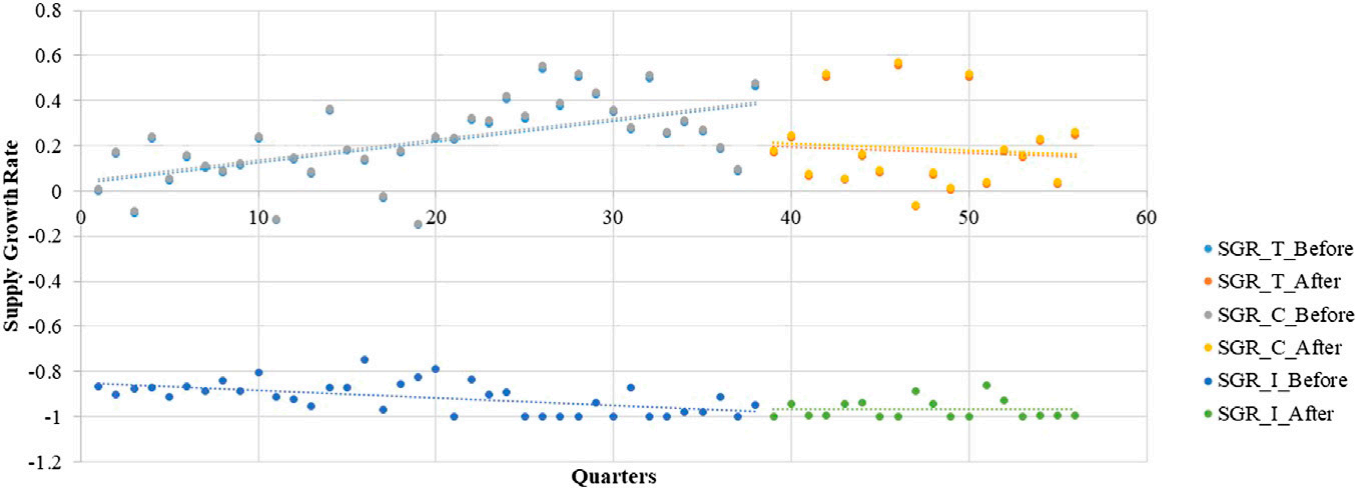
Segmented regression model showing LPCMs’ supply growth rates from 2005 to 2018.

During the pre-intervention period, the supply growth rate increased significantly in the total group (*β*_*1*_ = 0.0088, *p* < 0.001) as well as the continuous-supply subgroup (*β*_*1*_ = 0.0089, *p* < 0.001; Table 3). However, after the implementation of the LPMP, the LPCM supply in the total group (trend coefficient: *β*_*3*_ *=* −0.0132, *p* < 0.01; level coefficient: *β*_*2*_ = −0.1510, *p* < 0.05) and the continuous-supply subgroup (trend coefficient: *β*_*3*_ = −0.0133, *p* < 0.01; level coefficient: *β*_*2*_ = −0.1520, *p* < 0.05) shows a decreasing trend. For the intermittent-supply subgroup, the effect of the LPMP is not significant (Figure 3 and Table 3).

**TABLE 3:** Estimated coefficients of segmented regression models for the LPCMs supply growth rate before and after the LPMP from 2005 to 2018 (*N* = 56 quarters).

Parameter	Total group		Continuous supply subgroup		Intermittent supply subgroup	
	β	RSE	β	RSE	β	RSE
Pre-intervention slope	0.0088^***^	4.88	0.0089^***^	4.90	−0.0034^***^	−5.49
Intercept	0.0043	0.09	0.0117	0.23	−0.7930^***^	−31.57
Change in slope	−0.0132^**^	−3.36	−0.0133^**^	−3.36	0.0031	1.73
Change in intercept	−0.1510^*^	−2.28	−0.1520^*^	−2.27	0.0073	0.34
Quarter1	Yes	Yes	Yes	Yes	Yes	Yes
Quarter2	Yes	Yes	Yes	Yes	Yes	Yes
Quarter3	Yes	Yes	Yes	Yes	Yes	Yes

RSE, robust standard error; LPCMs, low-price chemical medicines; LPMP, low-price medicine policy. Two-tailed p value: *p < 0.05, **p < 0.01, ***p < 0.001.

### Influence on the Number of Supplied Medicines

Poisson regression shows that the number of supplied medicines changes over time. Table 4 shows a negative and significantly decreasing trend in the number of supplies from quarter to quarter before the intervention. The post-intervention period witnessed a significant decrease in the regression slope and level for medicines in the total group and continuous-supply subgroup (trend coefficient: *β*_*3*_ = −0.0027, *p* < 0.001; trend coefficient: *β*_*3*_ = −0.0060, *p* < 0.001).

**TABLE 4:** Estimated coefficients of segmented regression models for the LPCMs supply number before and after the LPMP from 2005 to 2018 (*N* = 56 quarters).

Parameter	Total group		Continuous supply subgroup		Intermittent supply subgroup	
	β	RSE	β	RSE	β	RSE
Pre-intervention slope	−0.0061^***^	−22.93	−0.0035^***^	−12.79	−0.0582^***^	−34.90
Intercept	4.8420^***^	754.47	4.7350^***^	853.54	2.7910^***^	59.92
Change in slope	−0.0027^***^	−4.94	−0.0060^***^	−9.21	0.0715^***^	12.19
Change in intercept	−0.0584^***^	−6.63	−0.0742^***^	−7.59	0.1740	1.92
Quarter1	Yes	Yes	Yes	Yes	Yes	Yes
Quarter2	Yes	Yes	Yes	Yes	Yes	Yes
Quarter3	Yes	Yes	Yes	Yes	Yes	Yes

RSE, robust standard error; LPCMs, low-price chemical medicines; LPMP, low-price medicine policy. Two-tailed p value: *p < 0.05, **p < 0.01, ***p < 0.001.

**FIGURE 4:**
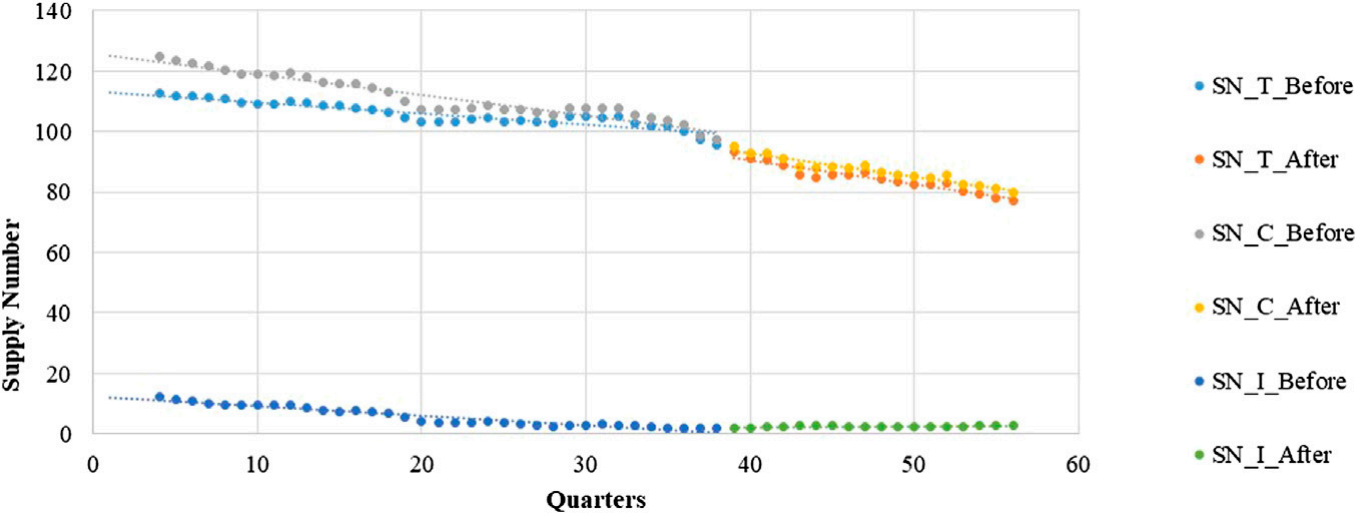
Segmented regression model showing LPCMs’ supply numbers from 2005 to 2018.

The intermittent-supply subgroup yielded different results. The pre-intervention period shows a significantly decreasing (β1 = −0.0582, *p* < 0.001) trend in the number of supplied medicines. After the intervention, however, a significant increase in the regression slope (β3 = 0.0715, *p* < 0.001) is noted (Figure 4 and Table 4).

## Discussion

This study identified the effects of the LPMP on LPCM supply in China. Our results show that the LPMP could not stop, but may have indeed caused, a decrease in both supply growth rate and number of LPCMs supplied in the continuous-supply subgroup. The LPMP has not raised production enthusiasm for LPCMs, but has triggered a switchover to other medicines. However, for the intermittent-supply subgroup, where medicine shortage has attracted public attention, both supply growth rate and number of supplies tend to stop the decreasing trend after the intervention.

Note that the period studied cover two interventions that cannot be distinguished. The 2015 price reform and the LPMP policy overlap each other. Since all LPMs are included in the UEBMI list, the 2014 LPMP can be regarded as a pilot initiative for the 2015 price reform. Moreover, since only LPMs were discussed in our study, the intervention year can be regarded as 2014. Thus, we cannot exactly evaluate the latest round of price reforms launched in 2015, since our study did not cover other medicines. However, the latest pricing reform could affect the supply of LPCMs, as explained below in more detail.

For the continuously supplied LPCMs in the market, the growth rate of supplied medicines presented a gradually increasing trend before the LPMP intervention. This could possibly be explained by a few factors. First, the population of China increased by approximately 8% during the statistical period, and more medicines needed to be produced (National Bureau Statistics, 2019). Thus, the supply growth rate increased before the intervention. However. the growth rate slope changes from 0.0089 before the intervention to -0.0044 after the policy, and the increasing trend changes to a decreasing trend. This shows a clear decline in production. We also find an accelerated decrease in the number of supplies (the slope changes from −0.0035 to −0.0095). Possible reasons could include the following. On the one hand, the implementation of the LPMP also increased Active Pharmaceutical Ingredient (API) prices monopoly (Liu, 2018). The expectation was that after the medicine price deregulation policy, the retail prices of LPM will increase. API production companies may have increased their prices, raising the cost of LPMs. For example, because of monopoly power, the raw material price increased nearly 50 times for calcium gluconate injection and nearly a hundredfold for aspirin (China Economic Net, 2018; CPhI.CN, 2018). On the other hand, the tender bidding process used by each province to select medicine manufacturers for government-run healthcare institutions has not changed (Su and Zhu, 2017). As the original tender bidding process prefers medicines with low prices, the LPMP had a restricted effect. For these two reasons, the companies may not have benefited as expected. Pharmaceutical companies are still not sufficiently motivated to increase the supply of LPMs.

Furthermore, the number of continuously supplied LPCMs decreased significantly before the implementation of the LPMP. Since high-price medicines tend to gain popularity among doctors in China, who obtain a higher profit by prescribing such medicines for patients (Yu et al., 2010). Thus, the mark-up policy in China could easily lead to a benefit chain among doctors, pharmaceutical enterprises, and sales representatives (Yang et al., 2016). Production of LPMs cannot provide a competitive advantage for pharmaceutical enterprises in comparison with high-price medicines. This reason could explain why the number of supplied medicines shows a decreasing trend before the intervention. It is worth to note that the latest round of pharmaceutical price reform in China was introduced in 2015 (National Development and Reform Commission, 2015). The reform not only relaxed price regulation of LPCMs but also freed the prices of medicines included in the UEBMI list. This provided an opportunity for pharmaceutical companies to stop producing LPCMs and switch to medicines with higher profits. This explains why the number of drugs supply continues to decrease after the intervention.

The intermittent-supply subgroup merits a discussion because most of the medicines have captured public attention even though the number of supplied medicines is small. Specifically, seven out of nine medicines are for emergency use. Their production situation is quite different. Both number of medicines and growth rate decreased gradually before the intervention. Unlike in the continuous-supply subgroup, the growth rate decreased a little despite the increase in the Chinese population. We believe that this severe medicine shortage is attributable to the low profits of companies. For example, pyrazinamide, gliclazide, ciprofloxacin, and propranolol faced severe scarcity in China according to media reports (CCTV, 2011; China Development Gate, 2018). Our results show that the production growth rate stopped decreasing significantly after the intervention (the slope changed from −0.0034 to −0.0003), whereas the number of supplied medicines shows a significantly increasing trend after the LPMP (the slope changed from −0.058 to 0.013). We believe that this is because, in addition to establishing of the LPMP, the government selected several enterprises for fixed-point production of LPMs in critical shortage to alleviate the shortage situation (National Health Commission of the P.R.C., 2016).

The causes of medicine shortages are complex and diverse, as they are related to both the supply and demand sides (Mayer, 2012). The primary causes of medicine shortages in the United States include inadequacy of raw materials and decrease in the number of manufacturers, besides other factors that cause delay in or termination of medicine production (Rochon and Gurwitz, 2012; Rosoff et al., 2012). In the European market, the main causes are API shortage, Europe’s dependency on API production in Asia, tendering, and parallel trade (Pauwels et al., 2015). Based on the specific issues, these developed countries have taken different measures to overcome medicine shortages. For example, the United States government requires manufacturers to inform the Food and Drug Administration (FDA) of any “discontinuance, interruption, or adjustment in the manufacturing of a medicine product that might result in a shortage” (Food and Drug Administration, 2011). Recently, the FDA developed a comprehensive three-pronged approach focusing on preventing shortages, quickly identifying anticipated shortages, and responding by remedying the underlying problems to the extent possible when shortages arise (Food and Drug Administration, 2018). In France, the government has called on medicine manufacturers to create a list of major therapeutic medicines of interest to be covered by preventive measures. Further, this policy includes a ban on exports by wholesalers in case of any shortage risk (Roehr, 2011).

China’s intervention, unlike those methods, removed price regulations. This is because previous researches showed that pricing, which was too low to stimulate the producers to supply medicines, was the main reason for medicine shortage in China (Yang et al., 2016). One empirical study found that LPM prices did increase after the implementation of the LPMP (Guan et al., 2018). Another study focused on how the hospitals in one province purchased more LPCMs (both in volume and number) after the LPMP (Rong et al., 2020). Our research used national-level supply data; although it reflects the average effect on 31 provinces in China, there may be differences across different provinces. We have evaluated the LPMP from the point view of medicine supply.

As for the solutions to address the medicine shortage issue, several policies could be established. First, we need to ensure that companies could make profits rather than only focus on price control relaxation, which would not stimulate production enthusiasm in companies. As already discussed, the medicine market tends to consume high-price medicines. An incentive system to generate preference for LPMs in doctors should be established. This could be accomplished through medical insurance payment reform. The key to this reform is to shift from a post-payment system to a pre-payment system. A full pre-payment system would support hospitals with fixed total incomes, reduce service cost, improve the utilization rate of resources and promote a reasonable allocation of health resources, make the hospital cost conscious, and avoid unnecessary consumption costs. Since hospitals tend to prescribe LPMs, their demand should be guaranteed. To meet the demand, companies would produce more LPMs at a profit in a free market. Furthermore, a reasonable salary system should be established for doctors in China, whose salaries are relatively low. Low salary could increase the moral risk of doctors and, in turn, the cost of the whole medical system. Third, big data technology should be utilized to supervise the price and supply of the API. This would allow early warning of medicine shortage and reduction of API monopoly. Finally, replaceable medicine plans should be designed to solve the shortage problem. Replaceable medicine plans should refer to therapeutic substitution and contingency plans, which will promote sustainable supply chains of LPMs.

This study has two main strengths. First, it quantitatively assessed the LPMP effects from a supply perspective and chose the growth rate and the number of supplied medicines as indicators. These indicators reflect not only the medicine supply growth rate change but also the production strategies from a long-term perspective. Second, the data used in this study are nationally representative.

However, our results should be interpreted cautiously as the study has several limitations. First, the data collected from the CEIC database are at a macro level, and do not provide detailed medicinal information. In particular, they do not include the strength and preparation data. Thus, we cannot identify the LPMs accurately. Second, LPTCMs were not considered in this study. Therefore, the results of this study may not be generalizable to all LPMs in China. Finally, we cannot distinguish the effects of co-interventions such as the 2015 pricing reform and fixed-point production.

## Conclusion

In conclusion, we have evaluated the effect of LPMP on the supply of LPMs. We have identified decreasing trends in growth rates and numbers of supplied medicines after the LPMP. The policy did not achieve its original goal for most of the LPMs—to increase medicine supply. We also found some interesting results by separating the LPMs into two subgroups. In the severe shortage subgroup (the intermittent-supply subgroup), the supply growth rates and numbers of supplies stopped their decreasing trend, and supplies tended to stabilize at a certain level after the LPMP. Comprehensive medicine policies, rather than just price deregulation, should be introduced to alleviate medicine shortage in China.

## Data Availability

Publicly available datasets were analyzed in this study. This data can be found here: The data analyzed in this study were obtained from the CEIC. Requests to access these datasets should be directed to Caijun Yang at yangcj@xjtu.edu.cn.

## Footnotes

1 The mark-up policy allowed public health facilities in China to generate revenues and issue bonuses. In addition, the income of individual staff was directly linked to revenue generation. Thus, doctors preferred expensive medicines and poly-pharmacy.

2 China’s National Development and Reform Commission (NDRC), which oversaw pharmaceutical pricing previously, controlled the prices of all medicines covered by the Urban Employee Basic Medical Insurance (UEBMI) by setting a maximum retail price (i.e., the price cap) for each medicine.

3 The UEBMI system, which was established in 1998, is one of three basic medical insurance systems in China. Mandatory participation is planned for all employees in the formal sector. The other two government-run medical insurance programs in China’s universal basic medical insurance system are the Urban Residence Basic Medical Insurance (URBMI) and the New Rural Cooperative Medical Insurance (NRCMI). The reimbursement drug list for URBMI is almost the same as that of UEBMI, but with pediatric medicines added. To date, the NRCMI has no national reimbursement drug list, and every province develops its own drug list based on the UEBMI list.
